# Sorafenib in the Treatment of Advanced Hepatocellular Carcinoma

**DOI:** 10.4103/1319-3767.37808

**Published:** 2008-01

**Authors:** Ali Ben Mousa

**Affiliations:** Division of Gastroenterology and Hepatology, Department of Medicine, Riyadh Military Hospital, Riyadh, Saudi Arabia

**Keywords:** Hepatocellular carcinoma, kinase inhibitor, sorafenib

## Abstract

Management of advanced hepatocellular carcinoma (HCC) is still a challenge to physicians since these patients are not candidates for surgical or ablative therapy. The disease carries a very poor prognosis with an expected survival of 4–6 months. No chemotherapeutic agent has been proven to improve the clinical outcome in such patients. A multikinase inhibitor, sorafenib, previously tested and found effective in other solid tumors recently found to significantly improve survival in patients with advanced HCC. Sorafenib exerts its action through inhibition of several kinases involved in both tumour cell proliferation and angiogenesis. It was well tolerated at a dose of 400 mg twice daily and permanent discontinuation of the drug was rarely required.

Hepatocellular carcinoma (HCC) is the most common primary liver tumor responsible for about 90% of liver cancers. It is the fifth common cancer in the world, and the third most common cancer in adult male population and sixth in female population in Saudi Arabia. Approximately 600,000 new cases are diagnosed annually worldwide, and 290 new cases (5% of all cancer cases) were diagnosed in Saudi Arabia in 2002.[1,2]

Although early HCCs with single lesion less than 5 cm or multiple HCCs that are less than three in number and less than 3 cm each are becoming more successfully managed with different treatment modalities, including hepatic resection, ablative therapy (radiofrequency ablation and percutaneous ethanol injection) and orthotropic liver transplantation, management of advanced HCC remains a challenge to physicians. Advanced HCC carries a very poor prognosis with a mean survival of 4–6 months. Many chemotherapeutic agents have been tried, but none had shown a significant result of improved survival or quality of life.

Sorafenib^®^ is a multikinase inhibitor that has recently obtained Food and Drug Administration (FDA) approval for the treatment of advanced renal cell carcinoma (RCC). It has been tried in several solid tumors, including HCC. A recent phase III trial has shown that sorafenib significantly extends survival for patients with advanced HCC.

This review will highlight the role of sorafenib in the management of advanced HCC, its mechanism of action and the safety profile.

## SORAFENIB

Sorafenib tosylate is a small, orally available molecule (MW 637 g/mol) identified through serial modification of commercially available Raf kinase inhibitor, GK-00687. It has a multikinase inhibitory action, and through blockage of pathways involving these proteins, sorafenib has an anticancer activity against several solid tumors. Some of these effects have been proven in clinical trials while others are still under investigation.

Sorafenib is given at a dose of 400 mg twice daily with a mean bioavailability of 38–-49%. It is metabolized primarily in the liver by two pathways: oxidative metabolism mediated by cytochrome p450 (CYP3A4) and glucourinidation mediated by UGT1A9. The mean half life is 25–48 h. Steady-state plasma level is achieved within 7 days. No impact of renal function was seen on sorafenib steady state if creatinine clearance is ≥ 30 ml/min however, no data is available for patients with creatinine clearance < 30 ml/min or for those on dialysis.

Metabolism is not altered in patients with chronic liver disease (CLD) Child-Pugh (CP) classes A and B, while no data is available for CP class C patients. No dose adjustment is required for age, gender, body weight, and mild to moderate renal or hepatic impairment.[3,4]

## MECHANISM OF ACTION

Sorafenib is an inhibitor of several kinases involved in both tumor cell proliferation (tumor growth) and angiogenesis (tumor blood supply). These include Raf, VEGFR and PDGFR.[5]

Raf is serine/theonine kinase, which when activated by Ras (membrane localized protein) stimulates gene transcription in the nucleus, leading to a variety of tumor-promoting cellular effects.

VEGF is the primary mediator of both normal and tumor-associated angiogenesis. It exerts this effect through several mechanisms, including induction of endothelial cell division and migration, promotion of endothelial cell survival through protection from apoptosis, and reversal of endothelial cell aging.

VEGF interacts with receptors (VEGFR 1,2,3) present on the endothelial cell surface, which leads to autophosphorylation of intracellular receptor tyrosine kinase, and a cascade of downstream proteins is activated.

PDGF has its receptor on the surface of capillary endothelial cells. The binding of PDGF to the receptors has several effects on endothelial cell motility and apoptosis.

## CLINICAL USES

In December 2006, the FDA approved sorafenib for the treatment of advanced RCC after demonstrating a two-folds increase in progression-free survival in treated patients. Nine hundred and three patients with advanced RCC were randomized to sorafenib versus placebo. Stable disease was observed in 78% and 55% of patients on sorafenib and placebo, respectively. Sorafenib significantly prolonged median progression-free survival (24 weeks) as compared with placebo (12 weeks), *P* ≤ 0.01. Tumor shrinkage was observed in 74% of patients treated with sorafenib as compared to 20% of patients who received placebo.[6]

Sorafenib has been evaluated in phase I/II trials for the treatment of many other solid tumors. In phase II trial, Sorafenib had modest anticancer activity comparable to monotherapy with other targeted agents in patients with recurrent or metastatic squamous cell carcinoma of the head and neck or nasopharyngeal carcinoma.[7] Sorafenib combined with gefitinib is also well tolerated, with promising efficacy in patients with advanced non-small cell lung cancer.[8] Malignant melanoma, metastatic breast, prostate and pancreatic cancers might benefit from sorafenib, and many trials are focused towards evaluating its efficacy in these types of cancers.[9–11]

### Sorafenib for treatment of advanced hepatocellular carcinoma

The roles of tyrosine kinase-dependant growth factors (Raf, VEGF and PDGF) and extracellular signal-regulated kinase (ERK) and MEK in the development of HCC have been well established. They play an important role in tumor angiogenesis and tumor growth by promoting proliferation and inhibiting apoptosis of tumor cells. Blocking these pathways was found experimentally to result in favorable anticancer effects,[12,13] but this was not proven clinically till recently.

In phase II study of sorafenib on patients with advanced inoperable HCC and CP A or B CLD, 137 patients with no prior systemic treatment were treated with oral sorafenib 400 mg twice daily. Three (2.2%) patients achieved partial response, 8 (5.8%) had minor response and 46 (33.6%) had stable disease for at least 16 weeks. Median time to progression was 4.2 months, and median overall survival was 9.2 months.[14] There were no significant pharmacokinetic differences between patients with CP class A and B.

Sorafenib HCC Assessment Randomized Protocol (SHARP) is a phase III, multicenter, randomized, placebo-controlled trial. From March 2005, 602 patients with advanced HCC, who did not receive prior systemic therapy at sites in the United States, Europe, Australia and New Zealand, were randomized to receive sorafenib 400 mg twice daily or placebo. The primary objective of the study was to compare overall survival between patients administered sorafenib and placebo. Median survival was 10.7 months in sorafenib-treated patients as compared to 7.9 months in placebo-treated patients (HR = 0.69; *P* = 0.0006).[15] SHARP was halted earlier last year (2007) after having achieved primary survival endpoint. These results were presented at the 43^rd^ annual meeting of the American Society of Clinical Oncology (ASCO). There were no significant differences in serious adverse event rates between the two groups, with the most commonly observed serious adverse events in patients receiving sorafenib being diarrhea and hand-foot-skin reaction.

Based on these findings, researchers suggest that sorafenib should be the new reference standard of care for first-line treatment of HCC, because there are currently no therapies that could significantly improve survival in liver cancer, and the safety profile of this agent is well understood. Sorafenib is pending approval from FDA for the treatment of advanced HCC. Positive results of this trial will open avenues for future research on HCC management using monotherapy or a combination of anticancer agents targeting MEK/ERK signaling pathway and other kinases involved in tumor growth and angiogenesis.

## DRUG SAFETY

A majority of trials on sorafenib showed favorable safety profile, and it is well tolerated by most of the patients at a dose of 400 mg twice daily.

The side effects are mostly related to the inhibition of kinases in normal cells. Diarrhea and dermatological side effects were by far the commonest, which affected 30–40% of patients. Hand-foot-skin reaction is frequently reported (25–30%), whereas milder forms of skin involvement include seborrhoic dermatitis like rash, alopecia, stomatitis and erythema multiforme are less common.[16] Dermatological toxicity usually responds to topical therapies and/or dose modification. Permanent discontinuation of the drug is rarely required.

Other side effects include diarrhea (30%), fatigue (18%), hypertension (8–16%) and pancreatitis (≤1%).

Many laboratory abnormalities were observed during sorafenib therapy, including hypophosphatemia (45%), elevated lipase (41%) and amylase (30%), lymphopenia (23%), neutropenia (18%) and thrombocytopenia (12%). Rarely, these abnormalities are associated with major morbidity or fatality.

Sorafenib can increase the risk of bleeding, especially in patients taking warfarin, and close monitoring of INR is advised in these patients. An increased incidence of cardiac events (myocardial ischemia) was observed (2.9% in sorafenib group vs 0.4% in placebo).[6] Sorafenib should be avoided in pregnancy (category D) and lactating mothers.

## CONCLUSION

Sorafenib is a new multikinase inhibitor, which has been shown to exhibit a significant anticancer effect in the treatment of solid tumors. It has been approved for the treatment of advanced RCC, and was recently found to improve survival of patients with advanced HCC as compared to placebo when used as monotherapy. The drug is well tolerated and does not adversely affect the quality of life in these patients. Sorafenib harbors the potential to make a major revolution in the management of patients with advanced, inoperable HCC.
